# Evolutionary origin of a periodical mass‐flowering plant

**DOI:** 10.1002/ece3.4881

**Published:** 2019-04-09

**Authors:** Satoshi Kakishima, Yi‐shuo Liang, Takuro Ito, Tsung-Yu Aleck Yang, Pei‐Luen Lu, Yudai Okuyama, Mitsuyasu Hasebe, Jin Murata, Jin Yoshimura

**Affiliations:** ^1^ Graduate School of Science and Technology Shizuoka University Hamamatsu Japan; ^2^ Department of Botany National Museum of Nature and Science Tsukuba Japan; ^3^ Department of Life Science National Taiwan Normal University Taipei Taiwan, ROC; ^4^ State Key Laboratory of Systematic and Evolutionary Botany, Institute of Botany Chinese Academy of Sciences Beijing China; ^5^ United Graduate School of Agricultural Science Tokyo University of Agriculture and Technology Tokyo Japan; ^6^ Department of Biology, TNM Herbarium National Museum of Natural Science Taichung Taiwan, ROC; ^7^ Department of Life Science National Chung Hsing University Taichung Taiwan, ROC; ^8^ Department of Life Science National Taitung University Taitung Taiwan, ROC; ^9^ School of Life Science The Graduate University for Advanced Studies Myodaiji Japan; ^10^ Division of Evolutionary Biology National Institute for Basic Biology Myodaiji Japan; ^11^ Botanical Gardens, Graduate School of Science The University of Tokyo Tokyo Japan; ^12^ Department of Environmental and Forest Biology State University of New York College of Environmental Science and Forestry New York New York; ^13^ Marine Biosystems Research Center Chiba University Chiba Japan

**Keywords:** life history, mass flowering, monocarpic perennial, periodicity, polycarpic perennial, *Strobilanthes*

## Abstract

The evolutionary origin of periodical mass‐flowering plants (shortly periodical plants), exhibiting periodical mass flowering and death immediately after flowering, has not been demonstrated. Within the genus *Strobilanthes* (Acanthaceae), which includes more than 50 periodical species, *Strobilanthes flexicaulis* on Okinawa Island, Japan, flowers gregariously every 6 years. We investigated the life history of *S. flexicaulis* in other regions and that of closely related species together with their molecular phylogeny to reveal the evolutionary origin of periodical mass flowering. *S. flexicaulis* on Taiwan Island was found to be a polycarpic perennial with no mass flowering and, in the Yaeyama Islands, Japan, a monocarpic perennial with no mass flowering. Molecular phylogenetic analyses indicated that a polycarpic perennial was the ancestral state in this whole group including *S. flexicaulis* and the closely related species. No distinctive genetic differentiation was found in *S. flexicaulis* among all three life histories (polycarpic perennial, monocarpic perennial, and periodical plant). These results suggest that among *S. flexicaulis*, the periodical mass flowering on Okinawa Island had evolved from the polycarpic perennial on Taiwan Island via the monocarpic perennial in the Yaeyama Islands. Thus, the evolution of life histories could have taken at the level of local populations within a species.

## 1 INTRODUCTION

Periodical mass‐flowering plants (called shortly periodical plants), such as bamboos (subfamily Bambusoideae, Poaceae) and *Strobilanthes* (Acanthaceae), flower and die gregariously and periodically at certain intervals of longer than 2 years (120 years at most) (Janzen, 1976; McClure, 1966). These periodical mass‐flowering plants are referred to as periodical plants because their life cycles are similar to those of periodical cicadas, which exhibit periodical mass emergence at 13‐ or 17‐year intervals in North America (Alexander & Moore, 1962; Simon, 1988). These periodical organisms including periodical plants and cicadas are characterized by the following life history traits: periodicity (reproduction according to a cycle of longer than 2 years), mass emergence and mass flowering (almost all individuals in a population synchronously reproduce), and semelparity and monocarpy (one reproduction event per lifetime).

In plants, the intervals between the two mass‐flowering events are not regular in non‐periodical plants (Sakai et al., 1999; Vander Wall, 2002). Monocarpic perennials are also found in a wide range of families; many of them lack mass flowering (Young & Augspurger, 1991). Other than bamboos and *Strobilanthes*, monocarpic perennials with synchronized flowering have been reported in several groups such as *Cerberiopsis* in Apocynaceae (Burd, Read, Sanson, & Jaffré, 2006; Read, Sanson, Burd, & Jaffré, 2008), and *Tachigali *in Fabaceae (Forget, Kitajima, & Foster, 1999; Kitajima & Augspurger, 1989). If mass flowering occurs periodically (the fixed intervals), these plants should be categorized as periodical plants. However, whether their intervals are periodical or not is unknown.

The periodicity of synchronized flowering is also known in annuals and strict biennials (almost all individuals flower and die in the second year from germination) (Kelly, 1985). Annuals flower synchronously in the same season every year. Most individuals of several strict biennial species flower synchronously every 2 years (Kelly, 1985). Flowering of these annuals and biennials is induced by seasonal changes or individual size (Harper, 1977; Rees, Sheppard, Briese, & Mangel, 1999; Rose, Rees, & Grubb, 2002). In contrast, flowering of periodical plants is induced by time from germination, but not by seasonal changes or individual size (Janzen, 1976). It is indicated by transplantation or propagation of cutting that does not affect timing of flowering in periodical plants (Kakishima, Yoshimura, Murata, & Murata, 2011; Tanimoto & Kobayashi, 1998; Watanabe, Ueda, Manabe, & Akai, 1982). Therefore, periodicity of more than 2 years is unique and found only in periodical plants. Thus, the evolution of periodicity of more than 2 years is a key event in the evolution of periodical plants.

The definition of periodical plants used in this report is as follows: periodicity (more than 2 years fixed year intervals of flowering after germination), mass flowering (almost all individuals in a population synchronously flower) and monocarpy (one flowering event per lifetime). In this definition, monocarpic perennials with either mass flowering or fixed three‐or‐more year periodicity are not considered a periodical plant. We here exclude annuals and strict biennials from periodical plants, because of the lack of yearly counting mechanisms, as explained above.

The evolutionary origins of periodical organisms are still unknown, although several hypotheses have been proposed. In periodical cicadas, the acquisition of periodicity is suggested to have preceded the selection of 13‐ or 17‐year cycles (Ito et al., 2015; Sota et al., 2013; Tanaka, Yoshimura, Simon, Cooley, & Tainaka, 2009; Yoshimura, 1997). However, the evolutionary history of periodical cicadas has not been verified, because non‐periodical, closely related species have not been found. In bamboo, a theoretical study based on phylogenetic inference suggests that the length of mass‐flowering cycles (the interval between two consecutive mass‐flowering events) has been multiplied in several groups, for example, from 15‐year cycles to 30‐, 60‐, 120‐year ones (Veller, Nowak, & Davis, 2015). Although the molecular phylogeny suggests cycle elongation in bamboos, the assumed annual ancestral species is unknown. Not that the information of closely related non‐periodical species has not been enough to verify the ancestral state of these periodical organisms.

**Figure 1:**
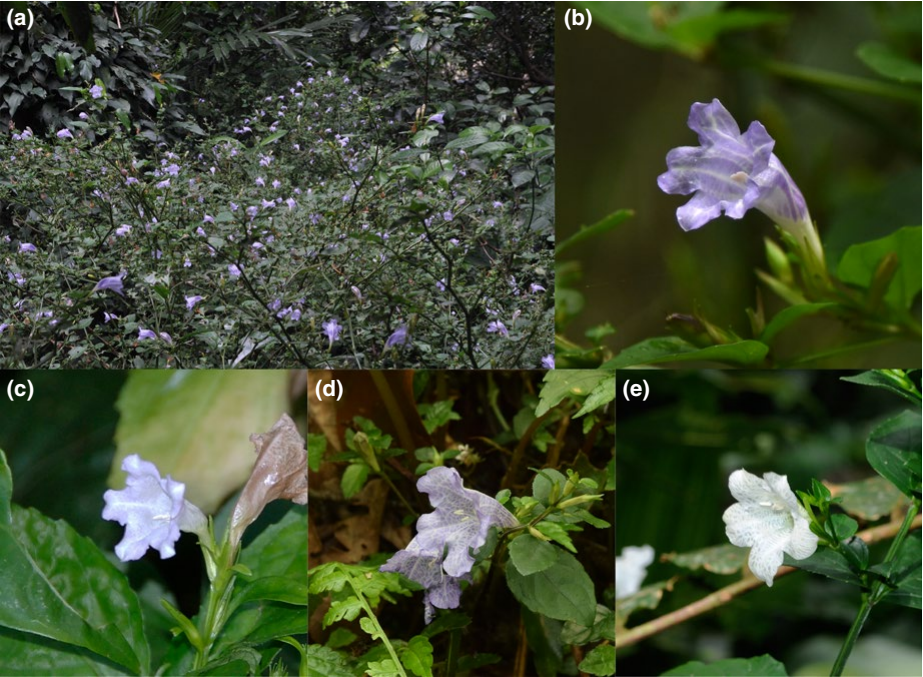
Mass flowering of *Strobilanthes flexicaulis* and the morphology of flowers. (a) Mass flowering of *S. flexicaulis *on Okinawa Island. The flowers of (b) *S. flexicaulis* on Okinawa Island, (c) *Strobilanthes tashiroi* on Okinawa Island, (d) *Strobilanthes rankanensis* on Taiwan Island, and (e) *Strobilanthes lanyuensis* on Lanyu Island

**Figure 2:**
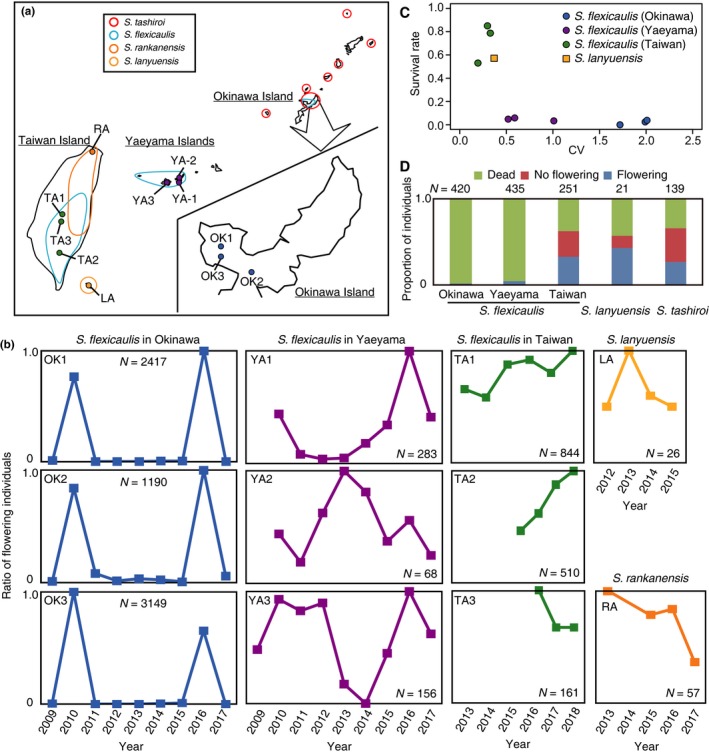
Distribution and life histories of *Strobilanthes flexicaulis* and relatives. (a) Distribution of four *Strobilanthes* species and the locations of the examined populations. Red, light blue, orange, and yellow lines show distribution of *S. tashiroi*, *S. flexicaulis*, *S. rankanensis,* and *S. lanyuensis*, respectively. Blue, purple, green, orange, and yellow solid circles show the locations of examined populations of *S. flexicaulis* on Okinawa Island, *S. flexicaulis* in the Yaeyama Islands, *S. flexicaulis* on Taiwan Island, *S. rankanensis* and *S. lanyuensis*, respectively. (b) Temporal dynamics of the ratio of flowering individuals calculated from the number of flowering individuals divided by the maximum number of flowering individuals during the surveyed period. OK1, OK2, and OK3 are Okinawa populations of *S. flexicaulis*. YA1, YA2, and YA3 are Yaeyama populations of *S. flexicaulis*. TA1, TA2, and TA3 are Taiwan populations of *S. flexicaulis*. LA is *S. lanyuensis*. RA is *S. rankanensis*. *N*: the number of examined individuals in each population. (c) CVs and survival rates. Blue, purple, and green circles show Okinawa, Yaeyama, and Taiwan populations of *S. flexicaulis*, respectively. A yellow square shows *S. lanyuensis*. (d) Consequences of the flowered individuals in the following year. Red, green, and blue show dead, survived with no flowers, and survived with flowers, respectively. The numbers above the bar indicate total sample sizes

To identify the evolutionary pathway from the ancestral state to the periodical mass flowering (emergence), the genus *Strobilanthes *is a prospective group with more than 50 periodical mass‐flowering species (Daniel, 2006; Janzen, 1976; Wood, 1994). *Strobilanthes* is distributed in East, Southeast and South Asia and includes approximately 400 species (Hu, Deng, & Wood, 2011). A molecular phylogenetic study indicated that periodical mass flowering has evolved several times independently in *Strobilanthes* (Moylan, Bennett, Carine, Olmstead, & Scotland, 2004). Here, we focus on *Strobilanthes flexicaulis*, a subshrub distributed in Japan (Okinawa Island and the Yaeyama Islands) and Taiwan (Taiwan Island) (Seok, Hsieh, & Murata, 2004; Wood & Scotland, 2003; Figures 1a,b and 2a). On Okinawa Island, this species exhibits periodicity by flowering gregariously every 6 years (Kakishima et al., 2011; Figure 1a). Almost all individuals of *S. flexicaulis* die after flowering and fruiting on Okinawa Island. The mass flowering in 2010 was common in all six surveyed populations on Okinawa Island.

*Strobilanthes flexicaulis* belongs to the Parachampionella group, which includes three other species (*S. rankanensis* and *S. lanyuensis* in Taiwan and *S. tashiroi* in Japan; Figures 1b–d and 2a) and records of one potential species in Sumatra and on Cocos Island (Wood & Scotland, 2003). *Strobilanthes tashiroi* is a polycarpic perennial (i.e., non‐periodical) herb endemic to the Ryukyu Islands, Japan, including Okinawa Island (Kakishima et al., 2011; Figures 1c and 2a). *Strobilanthes rankanensis *is a procumbent plant endemic to Taiwan Island (Hu et al., 2011; Figures 1d and 2a). *Strobilanthes lanyuensis* is morphologically similar to *S. flexicaulis* and endemic to Lanyu Island, Taiwan (Seok et al., 2004; Figures 1e and 2a).

In this report, we asked two questions: (a) What was the ancestral state of the periodical mass‐flowering *S. flexicaulis* on Okinawa Island? (b) How did the periodical mass flowering evolve from the ancestral state? We surveyed the overall distribution of the temporal dynamics of the number of flowering individuals for several years and the survival rates after flowering in the Parachampionella group by examining *S. flexicaulis* in Yaeyama and Taiwan Islands, *S. rankanensis,* and *S. lanyuensis*. We then investigated the molecular phylogeny of the Parachampionella group. Based on these data, we discuss the evolutionary pathway of the periodical mass‐flowering *S. flexicaulis* on Okinawa Island.

## 2 MATERIALS AND METHODS

### 2.1 Field observation of life histories

The number of flowering individuals of *S. flexicaulis*, *S. rankanensis,* and *S. lanyuensis* were examined in Japan and Taiwan. The three surveyed populations (OK1, OK2, and OK3) of *S. flexicaulis* on Okinawa Island were same as in a previous study (Kakishima et al., 2011; Figure 2a; Supporting Information Table S1). We also surveyed the number of flowering individuals of *S. flexicaulis* in three populations (TA1, TA2, and TA3) in the Yaeyama Islands and three populations (YA1, YA2, and YA3) in Taiwan Island (Figure 2a; Supporting Information Table S1). Field observations of *S. rankanensis *and *S. lanyuensis* were performed in one population (RA) on Taiwan Island and one population (LA) on Lanyu Island, respectively (Figure 2a; Supporting Information Table S1). In each population, we first fixed an observation area. The extant of the observation areas was shown in Supporting Information Table S1. Then, we counted all flowering (or fruiting) individuals in the observation area once in the flowering season per year. Even if we found the individuals with flowering buds or fruits but without flowers, these individuals were also treated as the flowering individuals. All flowering individuals were enumerated from 2009 to 2018. The flowering seasons of *S. flexicaulis* and *S. lanyuensis* were in winter (from November to the following April). When we observed flowering individuals from November to December, the records were treated as for the following year. For comparisons, the ratio of flowering individuals was calculated as the number of flowering individuals at each site divided by the maximum number during the observation period. The intensity of the mass flowering was measured by the CV (coefficient of variation) between years (Herrera, Jordano, Guitián, & Traveset, 1998; Kelly, 1994; Kelly & Sork, 2002; Silvertown, 1980; Webb & Kelly, 1993). We calculated the CV of *Strobilanthes* by *SD*/mean of the number of flowering individuals.

Monocarpic *S. flexicaulis* on Okinawa Island flowered in winter, bore fruits in spring, and then died in summer (Kakishima et al., 2011). To examine the number of flowering events in their lifetime (monocarpy or polycarpy), we labeled all flowering or fruiting individuals in the observation area of the surveyed populations every winter except the mass‐flowering years (Supporting Information Table S3). In the mass‐flowering years, we arbitrarily chose and labeled representative flowering or fruiting individuals in the observation area of each population on Okinawa Island because too many individuals were flowering (Supporting Information Table S3). The data of OK1–3 from 2009 to 2011 was already published in the previous paper (Kakishima et al., 2011). We checked whether the labeled individuals survived with flowers, survived without flowers or died in the flowering season of the following year (1 year after labeling) to examine whether they are monocarpy or polycarpy. We could not check the reproduction mode of *S. rankanensis* because the species is a procumbent plant and frequently propagates vegetatively; thus, it is impossible to examine whether the labeled individuals survived or died the year after flowering. Statistical analyses were performed using R for Mac OS X, version 3.4.1 (R Core Team, 2017). We used the generalized linear mixed model (glmer in the R package *lme4*; Bates, Maechler, Bolker, & Walker, 2015) using a binomial error distribution and logit link function to check the difference in survival rates among species and regions. For multiple comparisons, we used least square means (lsmeans in the R package *lsmeans*; Lenth, 2016).

### 2.2 DNA extraction and sequencing

Total genomic DNA was extracted from silica gel‐dried leaf tissue using the method using CTAB (Doyle & Doyle, 1987) with slight modifications after pretreatment with HEPES buffer (pH 8.0) (Setoguchi & Ohba, 1995). The sequences determined in this study were registered in the DNA Data Bank of Japan (DDBJ), which is linked to GenBank, and their accession numbers are provided with the sample information in Supporting Information Table S3.

The *PHOTOTROPIN2 *(*PHOT2*) gene was selected as a nuclear DNA (nDNA) marker because *PHOT2* has been successfully used to reconstruct the phylogeny of Verbenaceae in Lamiales, including Acanthaceae (Yuan & Olmstead, 2008). Phototropins are blue‐light receptors that control a range of responses that optimize the photosynthetic efficiency of plants (Christie, 2007). The fragments between exon 10 and exon 14 of the *PHOT2 *gene (five exons and four introns) were initially amplified by polymerase chain reaction (PCR) using the primers 10F and 14R (Yuan & Olmstead, 2008). PCR amplification was carried out using TaKaRa Ex Taq^®^ (TaKaRa Bio, Shiga, Japan) in a total volume of 30 µl. The PCR cycling conditions were an initial step for 2 min at 95°C, followed by 25 cycles of 45 s at 95°C, 45 s at 52°C, and 2.5 min at 72°C, with a final extension for 10 min at 72°C. The PCR products were purified using the GENECLEAN^®^ III Kit (BIO101, CA, USA). Purified DNA fragments were used as templates for the cycle‐sequencing reactions using the BigDye^®^ Terminator v3.1 Cycle Sequencing Kit (Applied Biosystems, CA, USA) with the same primers for PCR: 12F, 12R (Yuan & Olmstead, 2008), exon11F (ATGTAGATGAAGCTGTTCGAG), and intron12R (CATGTCAACAGTAGTTTGAGAG). The primers exon11F and intron12R1 were designed for this study. DNA sequencing was performed with an ABI PRISM^®^ 3130 DNA Sequencer (Applied Biosystems), and the obtained complementary sequences were assembled by Genetyx‐Mac/ATSQ Ver. 5.1 (GENETYX, Tokyo, Japan). When overlapping double peaks were found in the obtained electropherograms, we used the TA‐cloning system (Invitrogen, Carlsbad, CA, USA) for sequencing. At least 16 clones per sample were chosen and sequenced using the same procedure as in the first PCR, followed by direct sequencing. If the nucleotides in the cloned sequences were not detected by direct sequencing, they were considered PCR errors.

We sequenced three chloroplast DNA (cpDNA) regions: *trnS‐trnG*, *trnG*‐*trnR,* and *matK*. The fragments of *trnS‐trnG* were amplified by PCR using primers *trnS* (GCU) and *trnG *(UCC) (Hamilton, 1999). The PCR cycling conditions included an initial step for 2 min at 95°C, followed by 40 cycles of 45 s at 95°C, 45 s at 52°C, and 1 min at 60°C, with a final extension for 10 min at 60°C. Because this region was GC rich, the extension temperature was set lower than general conditions. The PCR products were purified with ExoSAP‐IT (USB, OH, USA) and directly sequenced with the same primers as used for PCR, and primer *trnS*1 (AATGTAAGGAGTCTGTCTTC) was designed for this study and used as necessary. The sequencing was performed in the same manner as nuclear DNA sequencing. Two other regions (*trnG*‐*trnR* and *matK*) were amplified by PCR using primers trnG_1F and trnR_22R for *trnG*‐*trnR* (Tripp, 2007) and matK‐AF and matK‐8R (Ooi, Endo, Yokoyama, & Murakami, 1995). The PCR cycling conditions were as follows: an initial step for 2 min at 95°C, followed by 40 cycles of 45 s at 95°C, 45 s at 52°C, and 1 min at 72°C, with a final extension for 10 min at 72°C.

### 2.3 Molecular phylogenetic analyses

Monophyly of the Parachampionella group, including *S. flexicaulis*, was supported in a previous study (Seok et al., 2004). Four *Strobilanthes* species from Japan and Taiwan were selected based on the previous study, and *S. formosana* and *S. dimorphotricha* were used as outgroup species (24 plant materials in total; Supporting Information Table S3). The sequences were aligned using ClustalW 1.8 (Thompson, Higgins, & Gibson, 1994) and then adjusted manually. The aligned lengths of the combined cpDNA sequences and the *PHOT2* sequences were 2,772 and 2,270 bp, respectively. The phylogenetic trees based on cpDNA and nDNA were constructed separately using a Bayesian approach with MrBayes 3.1.2 (Ronquist & Huelsenbeck, 2003) and maximum‐likelihood (ML) phylogenetic analysis using RAxML (Stamatakis, 2014). In the Bayesian phylogenetic analysis, the F81 model for nucleotide substitutions was selected for the tree based on the Bayesian information criterion (BIC) using the program Kakusan4 (Tanabe, 2011). Based on the selected model, we performed two separate runs of the Metropolis‐coupled Markov chain Monte Carlo (MCMCMC) analysis, each with a random starting tree and four chains (one cold and three hot). The MCMCMC was 10 million generations long, and the chain was sampled every 1,000th generation from the cold chain. The first 2,500 sample trees (25% of the total 10,000 sample trees) were discarded as burn‐in after checking that the average standard deviation of the split frequencies (ASDSF) reached a stationary state at <0.01. A 50% majority consensus tree of the output tree file from MrBayes was generated using FigTree ver. 1.3.1 (Rambaut, 2009). The ML phylogenetic analyses were implemented in RAxML 8 (Stamatakis, 2014) with a GTR+G likelihood model for nucleotide substitutions. The ML bootstrap proportions (BPs) and trees were obtained by simultaneously running rapid bootstrapping with 10,000 iterations followed by a search for the most likely tree. In the Bayesian analysis, the 50% majority‐rule consensus tree of all of the post‐burn‐in trees was depicted with Bayesian posterior probabilities (PPs). All the clades in the ML tree were recognized in the Bayesian tree; therefore, the BPs were plotted on the Bayesian trees. The statistically parsimonious networks were constructed using TCS version 1.21 (Clement, Posada, & Crandall, 2000). The incongruence length difference (ILD) test (Farris, Källersjö, Kluge, & Bult, 1994, 1995) was performed to check a significant incongruence between the molecular phylogenetic trees based on cpDNA and nDNA sequences.

## 3 RESULTS

Three *Strobilanthes flexicaulis* populations on Okinawa Island exhibited mass flowering in 2016, 6 years after the previous mass flowering in 2010 (Kakishima et al., 2011; Figure 2b; Supporting Information Table S4). In contrast, mass flowering was never observed in *S. flexicaulis* in the Yaeyama Islands and on Taiwan Island (Figure 2b). In the Yaeyama Islands, flowering was verified in 24 out of the cumulative total of 25 observation‐years for three populations (96%), while on Taiwan island, it was observed in 10 out of the cumulative total of 10 observation‐years for three populations (100%). CVs were high (1.72–2.01) in Okinawa populations exhibiting periodical mass flowering, whereas those in Taiwan populations were low (0.18–0.32) and those in Yaeyama populations were intermediate (0.52–1.01) (Figure 2c; Supporting Information Table S4). These results indicate that the intensity of mass flowering is very high in Okinawa populations.

In Okinawa and Yaeyama populations, most individuals of *S. flexicaulis* died after flowering and few survived in the next year (survival rates: 1.7%, 7 of 420 individuals in Okinawa populations; 4.4%, 19 of 435 individuals in Yaeyama populations), indicating that they were basically monocarpic (Figure 2c, Supporting Information Table S5). In Okinawa populations, those that flowered in mass‐flowering years all died (0%, 0 of 267 individuals); some of those that flowered in non‐mass‐flowering years survived the next year (4.7%, 7 of 153 individuals). All surviving individuals in both Okinawa and Yaeyama populations flowered and died in the following year. In Taiwan populations, in contrast, 62.9% (158 of 251 individuals) of flowering individuals survived the next year (Figure 2c, Supporting Information Table S5). Among 158 surviving individuals, 82 flowered again and the remaining 76 did not flower in the following year. For comparison of survival rates among “species” (*S. flexicaulis* on Okinawa Island, *S. flexicaulis* in the Yaeyama Islands, *S. flexicaulis* on Taiwan Island, *S. tashiroi* and *S. lanyuensis*), we fitted “species” as a fix term, and year and populations as random terms in the generalized linear mixed model. In this analysis, we found a significant difference among species (*N* = 1,310, *χ* ^2^ = 28.237, *df* = 4, *p* < 0.001). The survival rate of flowering individuals in Taiwan populations was significantly higher than that in Okinawa and Yaeyama populations (*p* < 0.001 and *p* < 0.001, respectively; Figure 2c,d) but was not significantly different from that of *S. tashiroi* (*p* = 0.771), indicating that they are polycarpic perennials. For *S. rankanensis* and *S. lanyuensis*, flowering individuals were observed every year during the surveyed periods (Figure 2b; Supporting Information Table S4), indicating no mass flowering. In the extremely rare *S. lanyuensis*, out of 21 individuals, 9 flowered again and 3 survived without flowers the next year (Figure 2d). The survival rate of *S. lanyuensis* was significantly higher than that of *S. flexicaulis* in Okinawa and Yaeyama populations (*p* < 0.001, *p* < 0.001, respectively) but was not significantly different from that of *S. flexicaulis* in Taiwan populations and *S. tashiroi* (*p* = 0.989, *p* = 0.992, respectively), indicating a polycarpic perennial. The survival rate of procumbent *S. rankanensis *cannot be measured because of vegetative propagation, although we confirmed that *S. rankanensis* was alive at the same place where flowering *S. rankanensis* had observed in the previous year.

**Figure 3:**
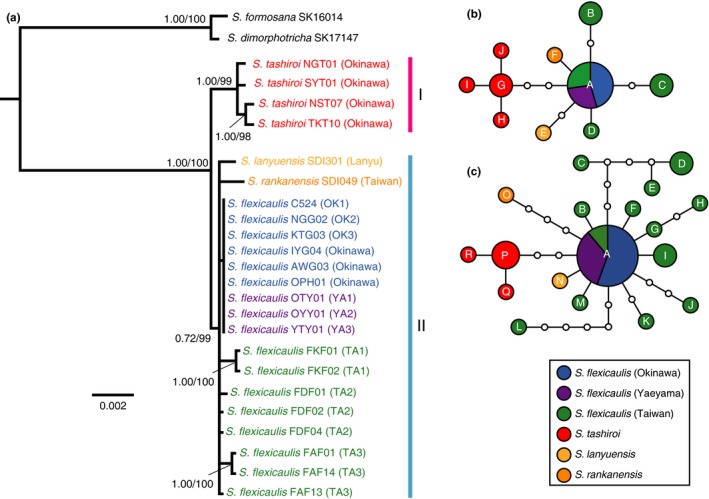
Bayesian molecular phylogenetic tree and haplotype networks. (a) A Bayesian molecular phylogenetic tree is based on combined sequences (cpDNA and nDNA). Bayesian posterior probabilities and ML bootstrap proportions are shown in each clade. Parsimonious networks are based on (b) cpDNA and (C) nDNA sequences

We reconstructed molecular phylogenetic trees within the monophyletic Parachampionella group (Seok et al., 2004) with two outgroup species based on three regions (*trnSG*, *matK*, *trnGR*) of cpDNA and one nDNA gene, *PHOT2*. The sequences of cpDNA and nDNA were combined because the ILD test (Farris, Källersjö, Kluge, & Bult, 1994, 1995) showed no significant incongruence between the molecular phylogenetic trees based on cpDNA and nDNA sequences (*p* = 1.000) (Supporting Information Figure S1a,b). A Bayesian tree (Figure 3a) and a ML tree (Supporting Information Figure S1c) based on combined sequences showed no clear incongruence. Both the Bayesian and ML analyses demonstrated that Clade I (*S. tashiroi*) was a sister to Clade II composed of *S. rankanensis*, *S. lanyuensis,* and *S. flexicaulis* with robust support (posterior probability/bootstrap proportion (PP/BP) = 1.00/99%), although the monophyly of clade II had only a weak support (0.72/99%) (Figure 3a). Within Clade II, both *S. rankanensis* and *S. lanyuensis* have species‐specific cpDNA haplotypes and nDNA allele types, indicating species‐specific differentiation. In Taiwan *S. flexicaulis*, four cpDNA haplotypes, and 13 nDNA allele types were recognized (Figure 3b,c). There was no clear geographical differentiation among *S. flexicaulis* of the three regions. *S. flexicaulis* in Okinawa and Yaeyama populations showed no sequence variations with the common cpDNA and nDNA types found in Taiwan populations (Figure 3b,c).

## 4 DISCUSSION

**Figure 4:**
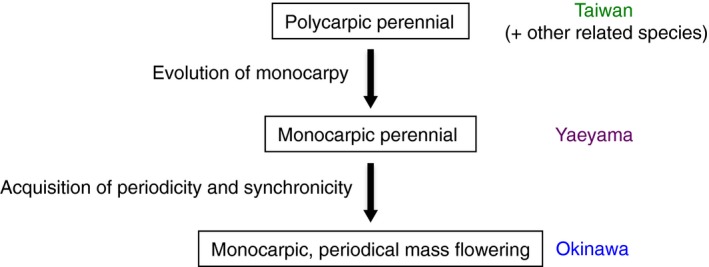
Schematic diagram of evolution of periodical mass flowering in *Strobilanthes flexicaulis*

We revealed that, among the Parachampionella group, *S. flexicaulis* on Okinawa Island is a periodical plant, *S. flexicaulis* in the Yaeyama Islands is a monocarpic perennial, and the remaining populations and species are polycarpic perennials (Figure 2). The ancestral life history of this group is suggested as characteristic of a polycarpic perennial because *S. tashiroi* (Clade I), which was a sister clade to that of the other species, are polycarpic perennials although statistical supports for Clade II are relatively low (0.72/99%) (Figures 2 and 3). Moreover, in population genetic point of view, it is indicated that Okinawa and Yaeyama populations of *S. flexicaulis* were derived from the Taiwan populations. This is because we found no genetic variation in both cpDNA and nDNA across *S. flexicaulis* in Okinawa and Yaeyama, and the cpDNA haplotype or the nDNA allele was shared with Taiwan populations, where we found much higher genetic variation (Figure 3b,c). These observations indicate that the periodical state of *S. flexicaulis* on Okinawa Island was derived from polycarpic states. Because the Yaeyama Islands are located between Taiwan Island and Okinawa Island (Figure 2a), we could reasonably infer that periodical *S. flexicaulis* on Okinawa Island evolved from a polycarpic perennial on Taiwan Island via a transient monocarpic perennial in the Yaeyama Islands (Figure 4). Thus, the evolution of periodical *S. flexicaulis* might have consisted of two steps: (a) the evolution of monocarpy in perennials and (b) the acquisition of periodicity and synchronicity.

The evolutionary acquisition of periodicity appeared in local populations on Okinawa Island of a single species *S. flexicaulis*. Both the final periodical state on Okinawa Island and the intermediate monocarpic perennial state in the Yaeyama Islands are found together with the ancestral polycarpic perennial state on Taiwan Island in a single species. No clear genetic differentiations in either the cpDNA or nDNA sequences are found among these three regions (Figure 3). The current findings make us hypothesize that both monocarpy and periodicity evolved recently via a change in a few genes. This hypothesis can be examined with comparing phenotypes, gene expression, and genomes among these closely related populations for the identification of the responsible genes (Ellegren et al., 2012).

Our findings, a periodical plant evolved from a polycarpic perennial via a monocarpic perennial, differ from the hypothesis in which the ancestral monocarpic annuals evolved into periodical plants via life cycle multiplication from one year to many years (Veller et al., 2015). The difference between our findings and the life cycle multiplication hypothesis proposed for Bamboos questions whether life cycle multiplication is responsible for the origin of periodicity or only for the elongation of life cycles once short cycle periodicity is established. Life cycle elongation by multiplication of intervals may have occurred after the evolution of periodical plants in *Strobilanthes* (Janzen, 1976). Different evolutionary mechanisms between Bamboos and *Strobilanthes* might have happened in the evolution of periodicity and life cycle elongation.

The several factors that might have derived the evolution of periodicity in *S. flexicaulis* can be considered. The cost of reproduction may play an important role in the evolution between polycarpy and monocarpy (Young & Augspurger, 1991). To verify the cost of reproduction, we need to examine the size of flowering individuals, the number of flowers and fruits per individual and the survival rates over individual lifetime. Two population‐level factors have been proposed to explain mass flowering (masting) in forests: pollination efficiency and predator satiation (Janzen, 1976; Kelly, 1994). These factors might have contributed to the evolution of mass flowering in *S. flexicaulis* on Okinawa Island (Kakishima et al., 2011). We need to examine further whether these factors are indeed working as selection on Okinawa Island, but not in the Yaeyama Islands and Taiwan Island. Future detailed study may elucidate the causal mechanisms underlying the evolution of periodical plants from polycarpic perennials via monocarpic perennials.

## CONFLICT OF INTEREST

None declared.

## AUTHOR CONTRIBUTIONS

SK, JM, and JY conceived the study. SK, YL, TYAY, and PL conducted the field works. SK conducted the laboratory works. SK, TI, and YO conducted the analyses. SK, YO, MH, JM, and JY wrote the manuscript. All authors read, revised, and approved the manuscript.

## Data Availability

DNA sequences: DDBJ accessions LC373925–LC373974, LC388312–LC388336, and LC384445–LC384477.
