# Supplementary material for: Evolutionary origin of a periodical mass‐flowering plant
Source: Ecol Evol. 2019 Apr 9;9(8):4373–81. doi: 10.1002/ece3.4881 (PMC6476870; doi:10.1002/ece3.4881)
Supplement: Supplementary file 1 [file ECE3-9-4373-s001.pdf]

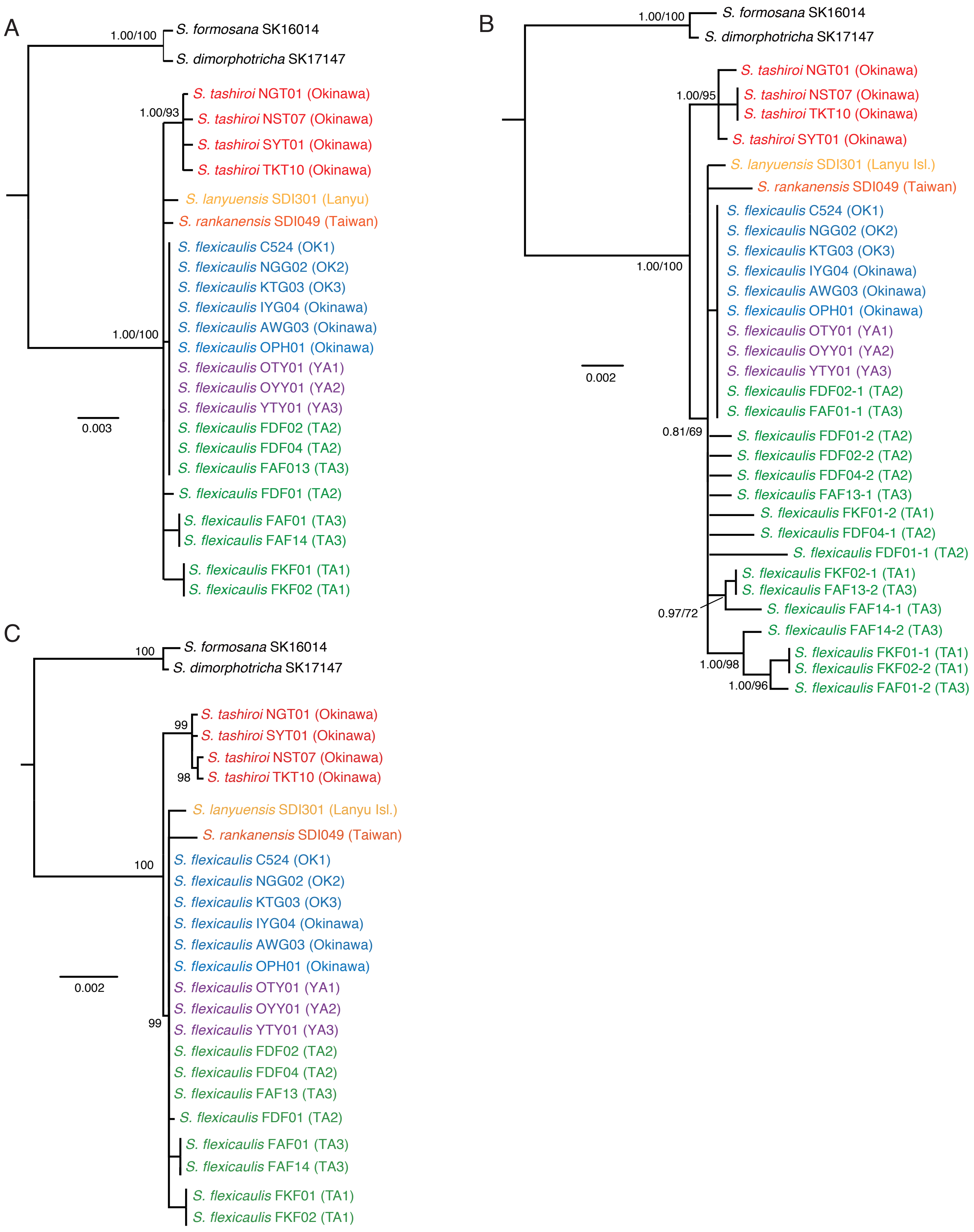

Fig. S1. Bayesian and ML molecular phylogenetic trees. (A) Bayesian molecular phylogenetic tree is based on cpDNA sequences. (B) Bayesian molecular phylogenetic tree is based on nDNA sequences. (C) ML molecular phylogenetic tree is based on combined sequences (cpDNA and nDNA). (A-C) Bayesian posterior probabilities and ML bootstrap proportions are shown in each clade.

Table S1. Locations of study sites.

Population	Locality	Geographical coordinate (N/E)	Altitude (m)	Extent (m x m)
OK1	Mt. Yae, Okinawa Island, Japan	26.64°/127.92°	280	5 x 100
OK2	Mt. Nago, Okinawa Island, Japan	26.59°/128.01°	250	10 x 100
OK3	Mt. Katsuu, Okinawa Island, Japan	26.63°/127.94°	290–380	5 x 300
YA1	Takeda, Ishigaki Isl., the Yaeyama Islands, Japan	24.40°/124.18°	140	15 x 100
YA2	Yamahara, Ishigaki Isl., the Yaeyama Islands, Japan	24.44°/124.17°	70	10 x 30
YA3	Yutsun, Iriomote Isl., the Yaeyama Islands, Japan	24.37°/123.88°	270	5 x 200
TA1	Shitou, Taiwan Island, Taiwan	23.66°/120.80°	1300–1360	10 x 1000
TA2	Dahanshan, Taiwan Island, Taiwan	22.41°/120.75°	1470–1510	10 x 1500
TA3	Alishan, Taiwan Island, Taiwan	23.50°/120.78°	1790	5 x 150
LA	Lanyu Island, Taiwan	22.02°/121.57°	300–350	5 x 500
RA	New Taipei, Taiwan Island, Taiwan	22.02°/121.57°	800–850	10 x 500

Table S2. The number of labeled individuals.

Species	Islands	Site	Condition	2009	2010	2011	2012	2013	2014	2015	2016	Total
S. flexicaulis	Okinawa	OK1	labeled	9	102	1	0	0	3	4	45	164
			survive, flower	4	0	0	0	0	0	0	0	4
		OK2	labeled	6	30	45	8	20	19	4	45	177
			survive, flower	0	0	0	0	0	0	0	0	0
		OK3	labeled	11	21	0	0	0	9	14	24	79
			survive, flower	0	0	0	0	0	1	2	0	3
	Yaeyama	YA1	labeled	32	49	9	4	5	20	38	112	269
			survive, flower	0	1	0	0	1	0	2	4	8
		YA2	labeled	12	5	3	10	16	13	6	9	74
			survive, flower	0	0	0	0	0	3	0	0	3
		YA3	labeled	14	27	24	26	5	0	13	27	136
			survive, flower	2	6	0	0	0	0	0	0	8
	Taiwan	TA1	labeled	–	40	10	21	35	31	15	12	164
			survive, flower	–	10	0	2	8	13	6	3	42
			survive, no flower	–	11	0	7	8	9	7	3	45
		TA2	labeled	–	–	–	–	–	–	30	17	47
			survive, flower	–	–	–	–	–	–	17	4	21
			survive, no flower	–	–	–	–	–	–	8	8	16
		TA3	labeled	–	–	–	–	–	–	–	40	40
			survive, flower	–	–	–	–	–	–	–	19	19
			survive, no flower	–	–	–	–	–	–	–	15	15
S. lanyuensis	Lanyu	LA	labeled	–	–	–	5	10	6	–	–	21
			survive, flower	–	–	–	3	3	3	–	–	9
			survive, no flower	–	–	–	0	2	1	–	–	3

Table S3. DNA types and accession numbers of examined samples.

Sample name	Taxon	Locality	cpDNA type	nDNA type	Accession numbers			
					trnSG	*trnGR*	*matK*	*PHOT2*
C524	*S. flexicaulis*	Mt. Yae, Okinawa Island, Japan (OK1)	A	A	LC373925	LC373950	LC388312	LC384445
NGG02	*S. flexicaulis*	Mt. Nago, Okinawa Island, Japan (OK2)	A	A	LC373926	LC373951	LC388313	LC384446
KTG15	*S. flexicaulis*	Mt. Katsuu, Okinawa Island, Japan (OK3)	A	A	LC373927	LC373952	LC388314	LC384447
IYG04	*S. flexicaulis*	Mt. Iyu, Okinawa Isl., Japan	A	A	LC373928	LC373953	LC388315	LC384448
AWG03	*S. flexicaulis*	Awa, Okinawa Isl., Japan	A	A	LC373929	LC373954	LC388316	LC384449
OPH01	*S. flexicaulis*	Mt. Oppa, Okinawa Isl., Japan	A	A	LC373930	LC373955	LC388317	LC384450
OTY01	*S. flexicaulis*	Takeda, Ishigaki Isl., the Yaeyama Islands, Japan (YA1)	A	A	LC373931	LC373956	LC388318	LC384451
OYY01	*S. flexicaulis*	Yamahara, Ishigaki Isl., the Yaeyama Islands, Japan (YA2)	A	A	LC373932	LC373957	LC388319	LC384452
YTY01	*S. flexicaulis*	Yutsun, Iriomote Isl., the Yaeyama Islands, Japan (YA3)	A	A	LC373933	LC373958	LC388320	LC384453
FKF01	*S. flexicaulis*	Shitou, Taiwan Island, Taiwan (TA1)	C	D, J	LC373934	LC373959	LC388321	LC384454, LC384455
FKF02	*S. flexicaulis*	Shitou, Taiwan Island, Taiwan (TA1)	C	D, I	LC373935	LC373960	LC388322	LC384456, LC384457
FDF01	*S. flexicaulis*	Dahanshan, Taiwan Island, Taiwan (TA2)	D	B, L	LC373936	LC373961	LC388323	LC384458, LC384459
FDF02	*S. flexicaulis*	Dahanshan, Taiwan Island, Taiwan (TA2)	A	A, F	LC373937	LC373962	LC388324	LC384460, LC384461
FDF04	*S. flexicaulis*	Dahanshan, Taiwan Island, Taiwan (TA2)	A	K, M	LC373938	LC373963	LC388325	LC384462, LC384463
FAF01	*S. flexicaulis*	Alishan, Taiwan Island, Taiwan (TA3)	B	A, E	LC373939	LC373964	LC388326	LC384464, LC384465
FAF13	*S. flexicaulis*	Alishan, Taiwan Island, Taiwan (TA3)	A	G, I	LC373940	LC373965	LC388327	LC384466, LC384467
FAF14	*S. flexicaulis*	Alishan, Taiwan Island, Taiwan (TA3)	B	C, H	LC373941	LC373966	LC388328	LC384468, LC384469
SDI0301	*S. lanyuensis*	Cultivation in Botanical Garendens of the Univ. Tokyo	E	O	LC373942	LC373967	LC388329	LC384470
SDI0049	*S. rankanensis*	Cultivation in Botanical Garendens of the Univ. Tokyo	F	N	LC373943	LC373968	LC388330	LC384471
NGT01	*S. tashiroi*	Mt. Nago, Okinawa Isl., Japan	G	Q	LC373944	LC373969	LC388331	LC384472
NST07	*S. tashiroi*	Mt. Nishime, Okinawa Isl., Japan	H	P	LC373945	LC373970	LC388332	LC384473
SYT01	*S. tashiroi*	Mt. Shioya-fuji, Okinawa Isl., Japan	I	R	LC373946	LC373971	LC388333	LC384474
TKT10	*S. tashiroi*	Mt. Terukubi, Okinawa Isl., Japan	J	P	LC373947	LC373972	LC388334	LC384475
SK16014	*S. formosana*	Mingchi, Ilan, Taiwan Isl., Taiwan	-	-	LC373948	LC373973	LC388335	LC384476
SK17147	*S. dimorphotricha*	Dashuehshan, Taiwan Isl., Taiwan	-	-	LC373949	LC373974	LC388336	LC384477

The letters of cpDNA type and nDNA type correpond with those in the haplotype networks in Fig. 3.

Table S4. The number of flowering individuals.

Species	Region	Population	2009	2010	2011	2012	2013	2014	2015	2016	2017	2018	Total	Average	SD	CV
S. flexicaulis	Okinawa Island	OK1	9	1043	1	0	0	3	4	1357	0	-	2417	268.56	533.89	1.99
		OK2	6	484	45	8	20	14	4	577	32	-	1190	132.22	227.38	1.72
		OK3	11	1882	0	0	0	9	14	1233	0	-	3149	349.89	703.63	2.01
	Yaeyama Islands	YA1	-	49	9	4	5	20	38	112	46	-	283	35.38	35.89	1.01
		YA2	-	7	3	10	16	13	6	9	4	-	68	8.50	4.44	0.52
		YA3	14	27	24	26	5	0	13	29	18	-	156	17.33	10.20	0.59
	Taiwan Island	TA1	-	-	-	-	115	102	153	160	140	174	844	140.67	27.54	0.20
		TA2	-	-	-	-	-	-	80	107	151	172	510	127.50	41.67	0.33
		TA3	-	-	-	-	-	-	-	69	46	46	161	53.67	16.26	0.30
S. lanyuensis	Lanyu Island	LA	-	-	-	5	10	6	5	-	-	-	26	6.50	2.38	0.37
S. rankanensis	Taiwan Island	RA	-	-	-	-	19	-	15	16	7	-	57	14.25	5.12	0.36

Table S5. Survival rates after flowering.

Species	Islands	*N*	Flowering	No flowering	Dead	Survival rate
S. flexicaulis	Okinawa	420	7	0	413	0.017
	Yaeyama	435	19	0	416	0.044
	Taiwan	251	82	76	93	0.629
S. lanyuensis	Lanyu	21	9	3	9	0.571
S. tashiroi	Okinawa	139	37	55	47	0.662
